# Practical Management of Iron Overload Disorder (IOD) in Black Rhinoceros (BR; *Diceros bicornis*)

**DOI:** 10.3390/ani10111991

**Published:** 2020-10-29

**Authors:** Kathleen E. Sullivan, Natalie D. Mylniczenko, Steven E. Nelson, Brandy Coffin, Shana R. Lavin

**Affiliations:** Disney’s Animal Kingdom^®^, Animals, Science and Environment, Bay Lake, FL 32830, USA; Kathleen.E.Sullivan@Disney.com (K.E.S.); Natalie.Mylniczenko@Disney.com (N.D.M.); Steven.E.Nelson@Disney.com (S.E.N.J.); Brandy.Coffin@Disney.com (B.C.)

**Keywords:** chelation, ferritin, hemochromatosis, hemosiderosis, oxidative stress, phlebotomy, transferrin saturation

## Abstract

**Simple Summary:**

Black rhinoceros under human care are predisposed to Iron Overload Disorder that is unlike the hereditary condition seen in humans. We aim to address the black rhino caretaker community at multiple perspectives (keeper, curator, veterinarian, nutritionist, veterinary technician, and researcher) to describe approaches to Iron Overload Disorder in black rhinos and share learnings. This report includes sections on (1) background on how iron functions in comparative species and how Iron Overload Disorder appears to work in black rhinos, (2) practical recommendations for known diagnostics, (3) a brief review of current investigations on inflammatory and other potential biomarkers, (4) nutrition knowledge and advice as prevention, and (5) an overview of treatment options including information on chelation and details on performing large volume voluntary phlebotomy. The aim is to use evidence to support the successful management of this disorder to ensure optimal animal health, welfare, and longevity for a sustainable black rhinoceros population.

**Abstract:**

Critically endangered black rhinoceros (BR) under human care are predisposed to non-hemochromatosis Iron Overload Disorder (IOD). Over the last 30 years, BR have been documented with diseases that have either been induced by or exacerbated by IOD, prompting significant efforts to investigate and address this disorder. IOD is a multi-factorial chronic disease process requiring an evidence-based and integrative long-term approach. While research continues to elucidate the complexities of iron absorption, metabolism, and dysregulation in this species, preventive treatments are recommended and explained herein. The aim of this report is to highlight the accumulated evidence in nutrition, clinical medicine, and behavioral husbandry supporting the successful management of this disorder to ensure optimal animal health, welfare, and longevity for a sustainable black rhinoceros population.

## Table of Contents

**1.**  **Introduction**
  *1.1*. *Why and how do we know BR have problems accumulating iron?*
  *1.2*. *Why does iron overload matter to wellness?*
  *1.3*. *How does IOD work in BR?*
  *1.4*. *What health complications connected to iron are of concern?*
**2.**  **Evidence-Based Veterinary Practice: Technical and Clinical Aspects**
  *2.1*. *Clinical Signs*
  *2.2*. *Diagnostic Testing*
    2.2.1. Serum iron
    2.2.2. Total iron binding capacity (TIBC) and transferrin saturation
    2.2.3. Ferritin
  *2.3*. *Perspectives on Integrative Monitoring*
  *2.4*. *Inflammatory Biomarker Connections to IOD*
  *2.5*. *Recommendations for Diagnosis*
**3.**  **Evidence-Based Nutrition Practices**
  *3.1*. *Could we make the diet more like the wild?*
  *3.2*. *Has diet ever changed the impact of IOD?*
  *3.3*. *What are the best practices for feeding black rhinos?*
**4.**  **Treatment and Prevention**
  *4.1*. *Hematologic Sampling Recommendations*
  *4.2*. *Therapeutic Large Volume Phlebotomy*
  *4.3*. *Techniques for VTLVP*
  *4.4*. *Pharmacologic Chelation Therapy*
  *4.5*. *History of Synthetic Chelation for BR*
**5.**  **Animal Husbandry and Operant Conditioning Practices**
**6.**  **Conclusions**

## 1. Introduction

Black rhinoceroses (BR; *Diceros bicornis*) under human care are predisposed to non-hemochromatosis Iron Overload Disorder (IOD; see Section 1.3 How does IOD work in BR?) with laboratory and histopathologic evidence of cellular injury, necrosis, and clinical signs similar to human iron overload disorders [1,2]. BR are native to eastern and central Africa and are Critically Endangered [3]. Poaching has reduced the wild population by >90% since 1970, and ~240 individuals are managed under human care with ~87 individuals in North America [4]. Over the last 30 years, BR have been documented with diseases that have either been induced by or exacerbated by IOD, prompting significant efforts in diagnostic, treatment, and prevention strategies [2,5,6].

Iron overload is an abnormal and chronic imbalance of iron metabolism with iron accumulation occurring over the course of years, saturating iron transport proteins and leading to organ damage and failure (reviewed in [7]). BR can live many years with IOD and typically do not show overt signs of illness until late in disease progression resulting in a shortened life span and reduced fecundity in this endangered species [8]. A lack of acute clinical symptoms in this species is not an honest marker of animal health nor iron balance.

**Figure 1:**
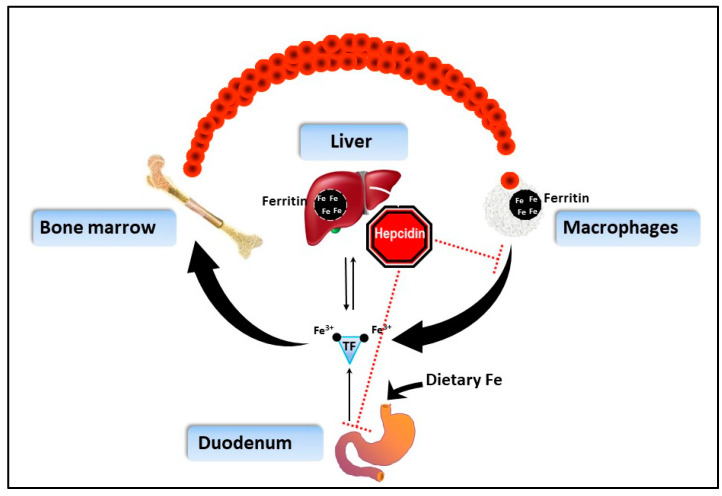
Regulation of iron homeostasis in humans (adapted and simplified from Knutson and Wessling-Resnick 2003) [10]. Dietary iron is absorbed in the duodenum, part of the small intestine. After regulated passage into the body, iron is transported primarily on transferrin (TF) to the bone marrow for red blood cell production. As red blood cells are broken down by phagocytic macrophages where iron is contained in ferritin (black circles), iron is primarily recycled to the bone marrow. Excess iron is stored in the liver, mainly within ferritin protein, until needed. Hepcidin, the iron regulating peptide hormone produced by the liver, blocks entry of iron from the small intestine and release of iron from macrophages by signaling the internalization of transport protein ferroportin. Once iron has entered the body, there is no route of excretion except forms of blood loss.

IOD is a multi-factorial disease process requiring an evidence-based and integrative approach for successful prevention and treatment. The aim of this report is to highlight accumulated evidence supporting the successful management of this disorder to ensure optimal animal health, welfare, and longevity. Specifically, as representatives of the Center for the Study of Iron in Rhinos (CSI-R), the authors will provide specific practical recommendations to treat this disorder in BR. Strategies and recommendations build on the collective expertise of colleagues and empirical data from experiences in BR nutrition, clinical veterinary medicine, husbandry, and operant conditioning. This report also integrates evidence from human medicine (Figure 1), as iron overload is a common clinical problem [9], and the management of the disorder has been studied extensively; thus, we use these learnings to supplement our strategies in BR under human care.

### 1.1. Why and How Do We Know BR Have Problems Accumulating Iron?

BR under human care are inevitably housed differently than their wild counterparts, which impacts homeostasis (i.e., physiological regulation and stability) through diet, environment, and management of social and behavioral interactions. The generally accepted etiology of IOD in BR is that the natural environment and diet of this browser species cannot be replicated. Additionally, there are possible genetic and physiological factors. Under managed care, BR consume more iron than would be available in the wild, and we cannot duplicate the complexity of African browse species, including chemical properties such as iron-binding capacity [11]. Furthermore, a predisposition to iron overload in BR may be due to genetic mutations affecting iron regulation and red blood cell (RBC) fragility [1,2,12,13,14,15,16]. Based on consistently elevated iron biomarkers and virtually every necropsy in BR under managed care in the past 60 years indicating moderate to massive iron deposition in multiple organs, BR are susceptible to excessive accumulation of iron [8]. Indeed, the amount of time under managed care correlated with iron accumulation in BR [1,14,15,16,17].

### 1.2. Why Does Iron Overload Matter to Wellness?

Iron plays an important role in free radical biology and pathology, which is a key factor in tissue damage in many pathological conditions [18]. Free iron is a catalyst for the formation of reactive oxygen species (ROS) such as peroxide and superoxide, causing inflammation and disease [19]. Excess iron increases the likelihood of infection and may reactivate dormant blood and tissue microbes that also cause inflammation [20]. Several factors affect iron signaling, including body iron load, circulating vs. stored iron concentrations, pathological conditions, and dietary habits [21]. Predicting outcomes and timelines is challenging, as the progression of IOD depends not only on the input of iron, but also on individual animal history, including pregnancy, parasite load, and confounding oxidative stressors, including those associated with metabolic syndrome.

### 1.3. How Does IOD Work in BR?

IOD does not manifest in a similar manner as hereditary hemochromatosis (HH) does in humans [22], and the terms IOD and hemochromatosis in BR often are used interchangeably, which is inaccurate. Instead of hemochromatosis, IOD in BR appears comparable to a compilation of multiple other forms of iron overload in humans (e.g., transfusion or thalassemia linked iron overload or RBC disorders) [23,24], via intoxication of iron into the body. The excessive iron, dietary in origin in BR, loads in tissues. Iron is primarily loaded in the BR spleen, liver, bone marrow, small intestine, and lung tissues [1,2]. Iron in excess can result in damage to these tissues, inflammation, and immune responses [8]. While the high iron levels from diets under human care (see Section 3. Evidence-Based Nutrition Practices) is a known contributor to loading, any potential malfunction(s) of known hormonal and genetic regulators of iron overload are unclear in BR [1,12,14].

On the other hand, HH is a human disease, not dietary in origin. Instead, HH and related variants are the result of particular mutations of genes causing defects in the iron control system known as the hepcidin/ferroportin iron regulatory axis [24]. HH shows iron deposition in primarily liver like BR, but also heavily in pancreas and heart tissues [10]. IOD in BR also appears to differ from HH as the iron-regulating hormone hepcidin shows a degree of functionality in BR as evidenced by histological evaluation of where iron is loaded in the liver (i.e., macrophages instead of hepatocyte cells) [1,23]. In HH, macrophages do not load iron due to hepcidin dysfunction; hepcidin dynamics in BR have not been evaluated yet due to assay challenges (see Section 2.3. Perspectives on Integrative Monitoring). Many factors, however, can influence hepcidin regulation of iron loading in vivo, including RBC turnover rates, kidney function (erythropoietin), and dietary iron bioavailability [23].

When necropsies are conducted in BR, hemosiderin deposits are evident in tissues (secondary iron overload), especially the liver (defined as multi-organ hemosiderosis); [2,25]. Hemosiderin is created in iron overload when ferritin, the iron storage protein, is damaged in a cell and abnormally metabolized for deposition [26,27,28]. Hemosiderin is also formed through complex physiological processes involving heme breakdown and iron storage [26]. Ferritin is shaped like a tiny (nano) cage which captures and holds iron (typically ~2000 atoms) [27,28]. When it takes on too much iron (>4000 atoms) when there is excess, the cage can expand and be altered to both become stuck in place (insoluble) and expose iron to create damaging reactions (reactive oxidant chemistry) [27,28]. An epidemiological investigation of BR iron status is required to paint a comprehensive picture of disorder machinations and progression. Such an investigation is difficult and technically not feasible in this species without correlations of antemortem (before death) liver iron concentrations and serum iron measures with post-mortem findings.

### 1.4. What Health Complications Connected to Iron Are of Concern?

Regardless of husbandry, history, pedigree, and/or seemingly normal complete blood count and blood chemistry profile (which does not include an iron panel), an animal with too much iron is prone to oxidative stress, inflammation, tumor formation, and infection (reviewed in [19,29,30]). The presence of hemosiderin is abnormal and is a contributing factor in the occurrence of many diseases across taxa. Deposits of hemosiderin in tissue are damaging to the function of that organ, and elevated iron leads to a myriad of health problems, including bacterial gum disease [31,32,33], insulin resistance [34,35], neurologic disease (reviewed in [36,37]), *Salmonella* infection [38], *Toxoplasma gondii* infection [39], inflammatory bowel disease [40], and ulcerative skin disease [41]. Furthermore, excess iron and hemosiderosis have been implicated in increased susceptibility to septicemia in a variety of taxa [42,43]. Multiple studies of necropsies and serum iron, transferrin saturation, and ferritin in BR (defined in Section 2. Diagnostic Testing) corroborate significant multi-organ iron deposits (reviewed in [2]) contributing to high morbidity and associated mortality in this species [2,6,15,44]. Further study is warranted to investigate the connections between the presence of high levels of circulating iron via IOD and BR disease and pathologies with known iron connections.

## 2. Evidence-Based Veterinary Practice: Technical and Clinical Aspects

### 2.1. Clinical Signs

Signs of iron overload are generic and can include lethargy, decreased appetite, reproductive depression, and behavioral changes. Animals are prone to comorbidities (simultaneous diseases), and IOD can be undetected in some animals until post-mortem examination or hidden because of secondary disease issues. Additionally, some believe that iron overload is not a significant threat as it does not cause overt or seemingly acute disease, as hemochromatosis does with obvious acute dysfunctions, such as hepatopathy, defined as an abnormal or diseased state of the liver. Iron overload is much more subtle at the onset and is more pervasive as overload progresses.

### 2.2. Diagnostic Testing

**Figure 2:**
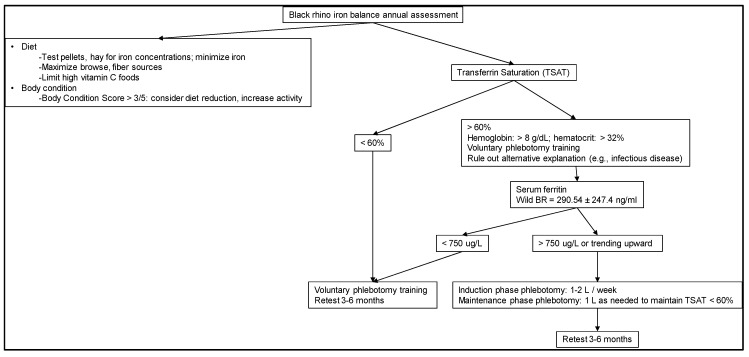
Decision Tree for approaching iron overload disorder (IOD) assessment in Black rhinoceros (BR) under human care. Serum ferritin in wild BR are from Miller et al. 2016 [47].

There is no single definitive test for iron overload in rhinoceros. Instead, there is a combination of serum biochemical markers that are shown to be appropriate tools, which give us information about individual animals and their general iron status [45] (Figure 2). Necropsy results have helped validate the use of these tests (reviewed in [2,8]). This strategy holds true in other species as well, including humans, where the details of iron metabolism and disease are well-studied [18]. It is important to have these applicable and useful measurements for a meaningful clinical assessment, and thus documentation of iron loading and general inflammatory state. The following parameters, in combination and with clinical context, allow us to evaluate an animal over time and observe trends in iron saturation status:

1. Serum iron (the amount of unbound iron in the blood)
2. Total iron-binding capacity (TIBC; indirect transferrin level)
3. Transferrin saturation (the percentage of TIBC occupied by iron) [46]
4. Ferritin (iron carrier protein; used with the exclusion of other diseases that may elevate ferritin)

#### 2.2.1. Serum Iron—Review and Comparative Evaluation

Iron is an important metal required by the body, but it is highly regulated and actively sequestered by cells to limit oxidative stress [48]. There is no process in animals to excrete iron; therefore, excesses remain and must be stored or contained (Figure 1) [49]. Higher than normal serum iron with low total iron-binding capacity (e.g., 100% saturation) indicates more iron is circulating in the body than can be contained.

Iron levels are abnormal when they reach or surpass the upper limit of the reference range in any group of animals. Serum iron values in wild BR (which we consider ‘normal’) were found to show a wide range of potential normality, 225.6 ± 106.7 µg/dL (*n* = 194) [47]. Serum iron values in BR under human care have a reported reference range of 204 ± 54.1 µg/dL (*n* = 374) [50], and from Kansas State University Diagnostic Lab, a reference range of 84–341 µg/dL has been reported (Kansas State Veterinary Diagnostic Laboratory; Manhattan, KS, USA). Alone, iron cannot be used to determine overload, but levels over the normal range should concern the clinician and prompt investigation into these other parameters. Elevated levels have been associated with overload as well as chronic liver disease [46].

Elevated iron suggests opportunities for chronic oxidative stress to organ systems. In excess, unbound iron is toxic and produces hydroxyl radicals and can damage cellular proteins, lipids, and nucleic acids directly [48,51], which can cause cellular, and thus, tissue damage. Iron also plays an important catalytic role in free radical pathology and oxidative damage that is observed in almost all major iron loaded and non-iron loaded diseases such as cardiovascular, neurodegenerative, hepatic, and renal diseases, as well as in cancer and aging [52]. With chronicity in humans, iron deposition (hemosiderosis or secondary iron overload) will occur in the heart, brain, kidney, liver, joints, skin, and endocrine system [49,53,54,55,56,57,58,59]. This chronic iron deposition in 60 BR necropsied was in the liver, spleen, bone marrow, and lungs, with some found in the small and large intestine, lymph nodes, and endocrine glands [2]. Lung involvement is unique to BR [1,37,58,60]. Hemosiderosis causes disease in several other species, but notably primates (e.g., callitrichids [61], lemurs [60]), birds [62], bats [63], and dolphins [64,65]) and is associated secondarily with other infectious and inflammatory diseases [66,67].

#### 2.2.2. Total Iron-binding Capacity (TIBC) and Transferrin Saturation—Review and Comparative Species Evaluation

Transferrin is the main protein that binds iron for transport and can be measured directly through immunochemical assays or indirectly through lower-cost colorimetric assays and molar calculation of TIBC; usually, the latter is used (TIBC) and reflects the amount of transferrin available to bind iron in circulation [68]. Under inflammatory conditions, this value decreases [69]. The UIBC (unsaturated iron-binding capacity) may also be determined as part of TIBC assessment methods. Recent work noted challenges with a methodology of assaying TIBC in BR involving pH shifts; however, different methods may have more success [70,71]. While there are a wide variety of methods possible for TIBC or transferrin, assessments validate the equivalence of either approach [68,72,73]. Alone, TIBC is not meaningful until compared to serum iron, where the ratio known as transferrin saturation or TSAT (100× serum iron/TIBC) corresponds to a saturation level that is a much better indicator of overload in the individual.

Typically, normal serum TSAT is 20–50% in mammals [42,43,46,74]. Johnson et al. [65] suggested that >60% is a level of concern for overload in dolphins, while in humans, anything >45% is a trigger [21,75]. In iron overload, TSAT is usually elevated >50% before serum ferritin consistently increases [76]. However, elevated levels of transferrin saturation are a clear indication of iron dysregulation and increased load. TSAT is a more sensitive indicator of initial iron loading than ferritin and given as a first-line test in humans but never evaluated in isolation [71]. It is also recommended to take blood samples before morning feeding, so serum iron is not impacted by iron diet intake, which can impact the TSAT [71].

#### 2.2.3. Ferritin

Iron is vital for life, and as mentioned above, it is critical to sequester iron when in excess; cellular ferritin performs that function of detoxification [77]. In fact, ferritin is solely an iron storage protein [49]. If iron were not present or available, ferritin levels could not be elevated, especially as there is a distinctly characterized genetic signaling pathway for regulating its production in each cell [78,79]. Hyperferritinemia in humans is defined by serum values >400 μg/L in males and >300 μg/L in females, but without clinical context, the value alone is difficult to interpret [80]. In BR, wild animal values are 290.5 ± 247.4 ng/mL [47]; therefore, consistently increasing or elevated values (e.g., >750 ng/mL; Figure 2) suggest an abnormal state. Ferritin itself is also a well-established marker of inflammation, and its dynamic nature shifts with non-iron storage-related diseases, sometimes resulting in episodic rather than chronic elevation [49]. However, given that there are no other diseases species-wide other than iron storage accounting for hyperferritinemia to the degree observed, it is very reasonable to weigh heavily on the chronic contribution of iron to elevated levels of ferritin as part of a holistic approach to IOD. Additionally, the continued upward rise of ferritin over time or a sustained elevated level is not likely with a fluctuating inflammatory condition such as a wound or infection. There is a limit on the production of ferritin (called apoferritin before iron is held within its nanocage structure), as a body can only produce so much of the protein at a time, so extremely high levels in circulation would indicate either a human error in measurement or the leakage of ferritin from iron-damaged cells [27]. This leakage is important as it further releases iron in an unbound form causing greater, continual damage [77]. Necropsies in BR show cellular injury and necrosis with evidence of released intracellular ferritin [2], which accounts for high serum ferritin concentrations, greatly exceeding apoferritin rates of synthesis [2,23]. While serum ferritin is not a perfect representation of iron stores, and hematologists in human iron studies have long worked to characterize iron carrier turnover and dynamics, ferritin and its measurement is still inextricably tied to further understanding iron overload.

The most practical and reliable ferritin test in BR is a commercially available equine assay (developed by [81]; available at the Kansas State Veterinary Diagnostic Laboratory (KSVDL). It has been shown to correlate well with BR ferritin in multiple studies [81,82]. Recent research has verified that although ferritin can vary across species, BR ferritin was found to be 91.4% identical to horse ferritin on a protein basis [23,83]. Based on the very high degree of homology between BR and horse L-ferritin genetically, it is likely that the polyclonal antibodies raised against horse L-ferritin will cross-react with BR ferritin, and the KSVDL assay is appropriate, and the data are valuable. The reference values the laboratory reports, however, are not ‘normal’, but rather represent all reported values from submissions in animals under human care, which are biased heavily with iron-overloaded animals.

### 2.3. Perspectives on Integrative Monitoring

The recent notion that ferritin should be a stand-alone diagnostic and that its value in monitoring IOD should be dismissed remains problematic [70,84,85]. In fact, in veterinary medicine, few diseases utilize a single serum biochemical marker for the diagnosis, let alone progression, of a disease state (e.g., renal disease). Changes in elevated ferritin (all cases >500 ng/mL) were considered not predictive of disease relative to onset of clinical signs in IOD cases; in those cases highlighted, multiple potential factors were involved (disease state, diet, environment, and unknowns) that understandably showed a variable pattern of ferritin across time [84]. Regardless, all elevated ferritin levels still indicated a state of iron overload, even if fluctuating but without the context of other holistic information, including TSAT or the ever-challenging antemortem liver biopsy. Herein, we have sufficient evidence-based information to support the continued use of ferritin, as outlined, as a part of a practical diagnostic and for monitoring of IOD in BR. It is important to characterize ferritin dynamics across time in the context of other assays.

In humans, there is considerable informed knowledge on iron metabolism and regulation, especially as more signaling molecules like hepcidin were discovered [86]. However, the overall basic monitoring strategy has not changed. A number of groups have attempted to measure BR hepcidin directly with no published success [87,88], and as noted previously, iron deposition in tissues demonstrates hepcidin has some degree of functionality in BR [1,23]. While the search remains for more informative diagnostics, including hyaluronic acid, micro RNA, microbial gut communities, and labile plasma iron measurement [89,90], none has shown clear promise as a new IOD marker beyond those that continue to be the benchmark currently across species. Therefore, continued monitoring of known direct iron-related panel parameters, including ferritin and transferrin saturation, is sensible. These tests are relatively inexpensive, and longitudinal information on individuals will demonstrate and elucidate patterns of loading.

Several other diagnostic tools have been used in the evaluation of iron disease or patient status affected by iron disease. Biopsies for histology and tissue iron concentration have been used in many species but require invasive and technically-challenging procedures to obtain samples antemortem. In the case of liver iron concentration, a larger piece of tissue is required than feasible to collect from BR unless post-mortem. Liver iron concentration has also fallen out of favor in human medicine due to the risk of complications [91].

### 2.4. Inflammatory Biomarker Connections to IOD

Oxidative damage and inflammation, which is inevitable with elevated iron, has had the attention of many rhinoceros researchers [70,82,92]. While these markers also do not indicate IOD, they can imply the negative effects of IOD and may connect with disease monitoring. Biomarkers that show some statistical significance over ‘normal’ include serum amyloid A (SAA), tumor necrosis factor α (TNFα), glucose to insulin ratios [82], superoxide dismutase, glutathione, and reactive oxygen metabolites in serum measured via electrophoresis (specifically α2 macroglobulin) [70,93]. Additional specific testable biomarkers include C-reactive protein (CRP), ceruloplasmin, and haptoglobin [94,95], but ceruloplasmin has not been formally tested, and haptoglobin has shown no obvious benefit in BR [81]. In assessing four animals across eight years at Disney’s Animal Kingdom^®^ (*n* = 91 samples/animal), correlations were poor between ferritin or transferrin saturation with ceruloplasmin and haptoglobin (0.000002 < R < 0.26), despite animals with well-documented IOD [45]. Some of these biomarkers may have utility in holistic patient assessment and may help provide a ‘bigger picture’ of total patient health but are not specific or sensitive enough to provide a diagnosis of IOD versus another inflammatory disease. While trend evaluation may be useful, relative values are not understood at this time. Additionally, these measurements do not allow immediate clinically useful information, and many may not be commercially available at this point. Another avenue of investigation based on some success in human medicine would be the impact of α-lipoic acid in protecting BR from the induced oxidative damage of iron overload [96,97]. While these assays are not the current solution to understanding IOD in BR, future research may circle back to how they are connected to iron accumulation, potentially as diagnostic technologies evolve.

### 2.5. Recommendations for Diagnosis

In summary, iron overload can be assumed with elevated serum ferritin levels when corroborated by elevated serum iron and transferrin saturation, especially when confirmed through long-term monitoring. As a general reference, average transferrin saturation and serum ferritin concentrations in free-ranging BR are 34% and 180 ng/mL, respectively [42]. Black rhino in a free-ranging situation tested with the same assay used by KSVDL in the US were also found to have serum iron ranging from 225.6 ± 106.7 µg/dL and ferritin values ranging 290.5 ± 247.4 ng/mL (*n* = 194) [47]. Further medical evaluation and testing and diet evaluation are recommended if transferrin saturation is elevated to >60% and serum ferritin concentrations are >750 ng/mL consistently, or if these values are trending upward (Figure 2).

## 3. Evidence-Based Nutrition Practices

Nutrition is an integral component of preventing IOD; thus, the assessment of dietary iron concentration and other nutritional factors is critical for iron balance in BR. BR likely evolved with a low-iron diet due to low iron concentrations (<215 ppm) [98] and limited availability of iron in leaves and stems of browse plants that contain high concentrations of iron-binding phenolic compounds [22,99]. The diet commonly offered to BR often has excessive iron concentrations (>300 ppm) as well as a poor representation of the nutrient and plant defense chemical compositions (e.g., polyphenols and alkaloids) found in wild browse [100]. The source of high iron concentrations in diets is pelleted feed, as dietary constituents and soil contamination can produce feeds high in iron, even when formulated to be low in iron (e.g., <200 mg/kg) [5,101,102,103,104]. It is not practical and may not be possible to provide a nutritionally complete diet under human care for BR without a pelleted energy source [45]. Furthermore, exhibit soil and vegetation can contain variable concentrations of iron, including those in excess to BR [5,105].

### 3.1. Could We Make the Diet More Like the Wild?

Studies on adding iron-binding compounds (e.g., polyphenols and concentrated tannins) to BR diets thus far lack compelling evidence to warrant inclusion, though no negative effects were found [106,107]. A better understanding of the biological activity of phenolic and tannin compounds is needed before routinely incorporating them. Naturally occurring iron-binding compounds such as those found in tea, grape pomace, quebracho, curcumin, etc., vary widely in composition and can bind other minerals such as zinc, copper, and calcium, further complicating nutrient balance. While supplemental iron-binding compounds and iron chelators can reduce iron absorption, the benefits should outweigh risks and must be considered with welfare and health outputs monitored. Assessing the nutrient bioavailability in vivo is invasive and obtaining total urinary and fecal iron output is challenging; these constraints hinder the development and subsequent inclusion of dietary iron-binding compounds in BR diets.

### 3.2. Has Diet Ever Changed the Impact of IOD?

**Figure 3:**
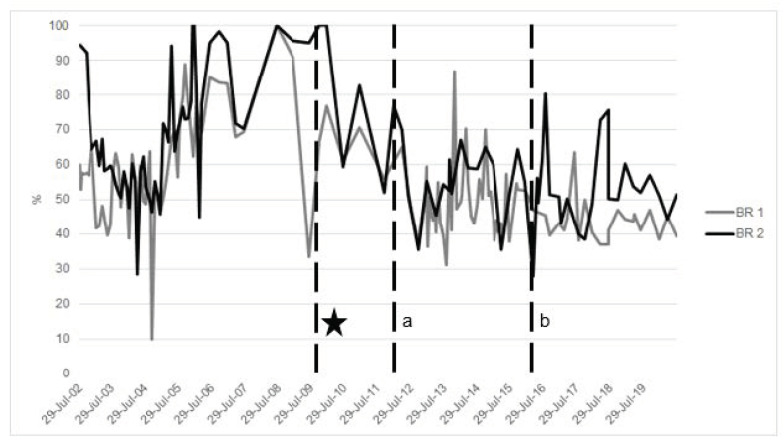
Transferrin Saturation (%) across time of two male black rhinoceros (BR1, BR2) at Disney’s Animal Kingdom^®^. * Major diet change occurred on 11 October 2009 for both animals. ^a^ Phlebotomy began on BR1—March 4, 2012; ^b^ Phlebotomy began on BR2—20 May 2016.

**Figure 4:**
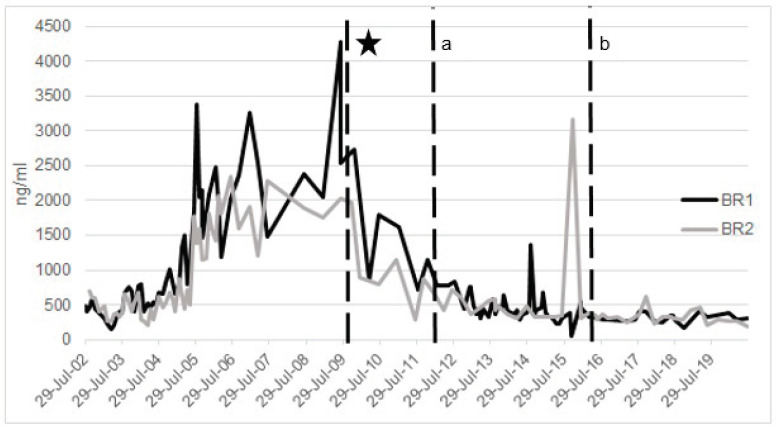
Serum ferritin (ng/mL) across time of two male black rhinoceros (BR1, BR2) at Disney’s Animal Kingdom^®^. * Major diet change occurred on 11 October 2009 for both animals. ^a^ Phlebotomy began on BR1—4 March 2012; ^b^ Phlebotomy began on BR2—20 May 2016.

There is evidence that a diet formulated to be low in iron can improve biomarkers of iron in BR. In particular, a reformulated pellet made by the Disney Animal Kingdom^®^ Nutrition team with Mazuri Exotic Animal Nutrition^®^, with decreased iron concentration from 772 mg/kg to 222–306 mg/kg, and a reduction of the amount of pellet offered to less than 30% of the total diet in four BR impacted biomarkers [45]. These nutritional modifications resulted in a total dietary iron concentration of 135 mg/kg. Thus, the majority of the diet was forage, with about 30% as fresh browse. The overall diet, including the reformulated pellet, was high in fiber (neutral detergent fiber (NDF) = 58.9%), and thus more consistent with a wild diet. The four BR consumed the reformulated diet, including two rhinos that were markedly iron-overloaded (ferritin >4000 ng/mL and transferrin saturation at 100%) [45]. Within a month post-diet change, serum ferritin decreased in all four animals, regardless of concomitant phlebotomy [50]. The other two younger animals showed reduced IOD biomarkers. Currently, both animals show no evidence of IOD compared to age-matched conspecifics under a more traditional diet. These results highlight the significant impact that diet modification can have on iron loading in the BR (Figure 3 and Figure 4).

### 3.3. What Are the Best Practices for Feeding Black Rhinos?

An appropriate diet for BR maximizes animal welfare, lifespan, and reproductive output. Recommendations for feeding must consider not only iron but also the many interconnected nutrients and metabolic concerns which may exacerbate iron-related issues, such as obesity. Based on empirical evidence, the following specific feeding guidelines for BR are recommended [50]:

- **Iron should be limited.** Iron concentration in the diet is recommended to not exceed 300 mg/kg dry matter (DM) or about 6 g of iron per day for a 1300 kg BR fed 1.5% BW in DM [5]. Based on the availability of feed items such as low-iron pelleted feed and browse (Table 1), a dietary iron concentration less than 300 mg/kg (dry matter basis) is a practical recommendation for BR [5,108]. Consider testing exhibit soil and vegetation to ensure they are not significant sources of iron in the diet of BRs [5,105].
- **Monitor individual body weight and body condition.** Feed 1–3% of body weight (BW) on an as fed basis, 1–2% on a dry matter basis. Maintaining appropriate body weight is critical as iron imbalance and obesity are presumed to be related; iron balance also is implicated with metabolic syndrome and associated negative health impacts [82]; however, the exact mechanism is not yet clear. Individual assessment of BR for bodyweight regularly across time (ideally weekly) and tracking with diet consumption is recommended [50,100]. Body condition scoring systems (e.g., a 1–5 scoring system based on ~seven body areas) are subjective, with varied recommendations on what is considered ideal depending on housing conditions along with medical and physiological considerations. Typically, a score of 3–4 out of a 5-point scale is considered ideal under human care. [109,110]. To optimize health, adjust diets as needed to ensure animals are within a target body weight range set for the individual animal.
- **Feed at least twice daily.** It is recommended to feed pellets in two feedings each day with forage to ensure maximum absorption of macronutrients [108]. Iron is not the only nutrient to consider in feeding complex diets to rhinos under human care. A single feeding would not be ideal for multiple reasons, including digestive efficiency, microbial community maintenance, satiety, and natural foraging behaviors.
- **Feed appropriate pelleted feeds**. The pellets milled for zoo animals vary widely in nutrient composition, and not all available pellets are appropriate for browsing species. Pellet formulations for BR are recommended to be high in fiber and low in starch and soluble sugars (Neutral Detergent Fiber (NDF) = 40–60%) [5]. Starch and sugar must be limited as these items can be associated with severe dental plaque, have metabolic impacts, and contribute to obesity [50,111,112]. A maximum of one-third of total calories is recommended to come from a pelleted concentrate. This limit avoids high pellet inclusion rates, which could be negative for dental health, body weight, and proper digestive health due to lack of long particle fibers [99].
- **Alfalfa hay should be limited**. This recommendation is due to its high protein, calcium, and iron, which can also create diarrhea and colic [5,50,108]. Conversely, low-quality hay (straw, wet/moldy, low nutrient content) is also not recommended due to the risk of intestinal impaction and/or colic. The iron in alfalfa is also held in a potentially highly bioavailable form (plant ferritin) [50].
- **Maximize browse and provide access to hay**. Preferably high quality roughage, ideally grass hay — not legume-based, as well as clean water and salt *ad libitum* [108]. Browse options may vary based on season and region, with options to freeze or ensile [110,113]. As browse best approximates the natural physical form of BR diets; it has the potential for iron-binding [114].
- **Total dietary vitamin E concentrations should be 150–200 IU/kg diet.** Extra supplementation may be necessary in addition to vitamin E in pelleted feed dependent on serum evaluation [17]. Vitamin E is a critical antioxidant that protects against ROS created by and including iron [50,115,116]. As BR lack some natural antioxidant production, ensuring dietary alpha-tocopherol (vitamin E) serves as a necessary preventive [50,115,116].
- **Phosphorus levels in the serum should be monitored and supplemented where appropriate.** BR have a predisposition to deficiency and continued concern for hemolytic issues; additionally, there is a link between phosphorus and iron metabolism [8,117]. Supplementation of monosodium phosphate and/or wheat bran in addition to phosphorus provided in a pelleted diet is recommended based on serum assessment. Naturally low phosphorus carriage in BR RBC (2–5% other mammals) [116] is thought to be connected to RBC fragility and potentially elevated RBC turnover [8,14,23]. In support of supplementation, higher levels of dietary phosphorus have been documented to combat anemic hemolytic crises in this species [8,23,117].
- **The calcium to phosphorus (Ca:P) ratio of the diet should be 2:1 (no less than 1:1).** A well-formulated pellet will provide appropriate calcium and phosphorus to meet the nutrient requirements of BR. An appropriate ratio eliminates the need for calcium supplementation, which can be contaminated with iron [102]. Grass hay typically is 1:1 and alfalfa 3:1, the latter of which can lead to hypercalcemia and hypophosphatemia. The amount of phosphorus added as a supplement should not unbalance the Ca:P ratio in the diet, in the amounts recommended based on body weight. The diet is balanced primarily with the pelleted portion, which is the majority of the dry matter of the diet and typically has the optimal 2:1 ratio (Table 1). Inverted serum Ca:P ratios are incredibly rare in rhinos; instead, hypercalcemia cases are far more common. As black rhinos physiologically appear to have an increased need for phosphorus, which is utilized for RBC turnover, they appear able to maintain serum Ca:P ratios of 2:1 despite a potential intake between 1:1 to 2:1.
- **Avoid non-specific mineral supplements and mineral salt blocks.** Plain salt blocks have minimal to no iron content and are appropriate [50].
- **Limit high vitamin C diet items (e.g., citrus fruits)**. Also, avoid feeding these foods at the same time as pellets due to increased iron availability in the presence of acidic foods such as vitamin C (Table 1) [118].
- **Training and enrichment diet items should be low in sugar, starch, and iron.** Target less than 10% of the total diet comprising of training and enrichment foods. Take into consideration high-sugar, high-starch, and high-iron items (such as molasses-based foods), which often are included in balanced diets for BR (Table 1) [50].

## 4. Treatment and Prevention

The timeframe for overload is gradual, highly variable, and determined by individual metabolism, iron intake, historical metabolism changes (e.g., pregnancy, an established iron sink), and other disease states. It can take years for iron to accumulate before any change is notable; onset is not predictable. When is it too late? The simple answer is ‘never’; treatment can always be initiated. However, the degree of overload will affect the results and the anticipation of when to see a change in serum biochemical parameters. If an animal is heavily overloaded, the time to see a difference may take months compared to an animal relatively with less iron. An expectation for an immediate drop in serum parameters with any method chosen is unrealistic. Active treatment involves minimizing dietary iron (see Section 3. Evidence-Based Nutrition Practices) and established methods to remove iron from any mammalian species; phlebotomy and chelation therapies. Prevention would be ideal, but maintenance is most likely given limitations of available diet and browse species, which provide a constant influx of iron.

### 4.1. Hematologic Sampling Recommendations

A comprehensive preventive health program for BR is recommended and, in addition to dietary management, includes routine hematologic sampling for continuous diagnostic evaluation (Figure 2, Figure 3 and Figure 4). Newly acquired animals to a facility should have a baseline sample collected and archived. Serial samples are important for monitoring trends; quarterly samples, as a recommended example, provide a robust series of data to review and to confirm or monitor IOD. At the very least, annual samples including iron panels as well as a minimum database (complete blood count and serum chemistry) should be conducted to be able to catch a change in biochemical parameters (e.g., normal hematocrit >32% and hemoglobin >8 g/dL) and organ function. These tests also help with a routine overall assessment of the animal. Management and treatment strategies will depend on how much the animal is iron-loaded (Figure 2).

### 4.2. Therapeutic Large Volume Phlebotomy

Therapeutic large volume phlebotomy (TLVP) is commonly used in human medicine [119,120,121,122], and it has proven effective in dogs where ferritin and hepatic iron were reduced with phlebotomy treatment [123] and in BR [45,110]. TLVP has been used at multiple institutions accredited by the Association of Zoos and Aquariums (AZA; e.g., Columbus Zoo and Aquarium [124], Denver Zoo [124,125], Milwaukee County Zoo [108,126]) and extensively at Rotterdam Zoo in the Netherlands [110]. In the authors’ experience, TLVP has shown to be a safe and effective strategy for not only reducing excess storage iron in overloaded patients but also maintaining normal iron homeostasis as animals age (Figure 3 and Figure 4). By combining an applied model for large volume blood collection in Atlantic bottlenose dolphins (*Tursiops truncatus*), and clinical models in humans with iron overload (Johnson et al., 2009; D. Paglia, 2004), parameters were extrapolated for voluntary TLVP (VLTVP) in BR at Disney’s Animal Kingdom^®^ (DAK) [127]. In dolphins, during the induction phase, 5–8 mL/kg (1–2 L) of blood was removed per weekly session with varying times for transition to a maintenance phase (often several months). In the maintenance phase, one liter was collected until transferrin saturation was <60% [65]. BR must participate in their treatments for success; thus, a training program (see Section 5. Animal Husbandry and Operant Conditioning Practices) must be built around this treatment modality.

Based on the above guidelines, an average BR (800–1300 kg) would require minimally 4–6.5 L of blood per session. While this is possible, it is often technically difficult to achieve. The blood volume goal, therefore, was revised to 2–4 L per session as improvement in blood values was still observed. More than 4 L in a 20–40 min period is not likely with only one venipuncture site, but it can be accomplished with two. While this volume seems low compared to other species relative to body mass, this strategy has proven successful. The frequency of phlebotomy is variable and dependent on the degree of overload; monitoring serum iron panels with each session will help determine the frequency and volume of phlebotomy. Monthly VTLVP can be accomplished. Due to our preventive strategy, IOD is well-managed in both rhinos, and animals need only quarterly phlebotomies based on serum biomarkers (BR 1 and 2; Table 2; Figure 3 and Figure 4). Weekly sessions are achievable; however, this strategy may hinder animal compliance. One successful strategy for maintenance of low overload has been a quarterly goal of 2–6 L (dependent on the degree of IOD) with sessions occurring as tolerated by the individual until the minimum volume is achieved within the quarter, regardless of the number of sessions.

### 4.3. Techniques for VTLVP

**Figure 5:**
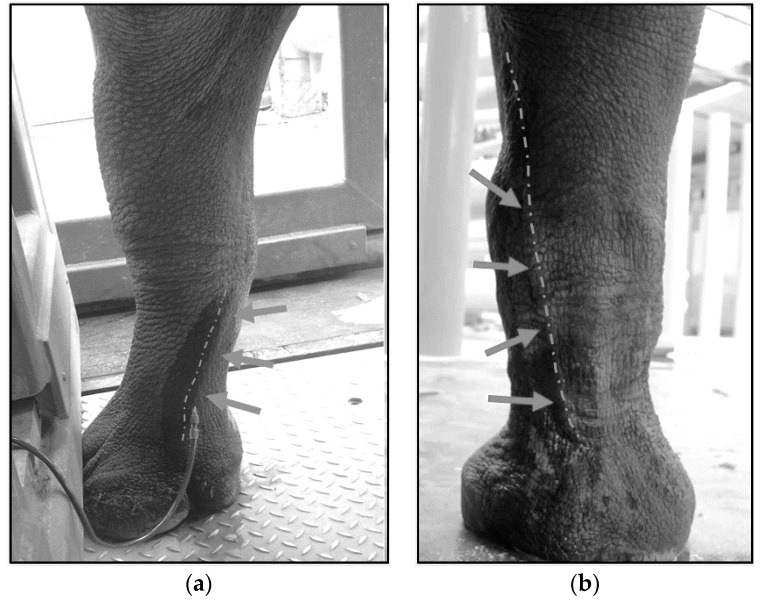
Phlebotomy Sites/Venous Access. Options for venipuncture sites with larger vessels that tolerate a large volume for blood collection include the metacarpal vein; (**a**) lower distal hindlimb or the radial vein; (**b**) medial forelimb crossing the carpus. Photos were taken in an off-exhibit animal holding area.

**Figure 6:**
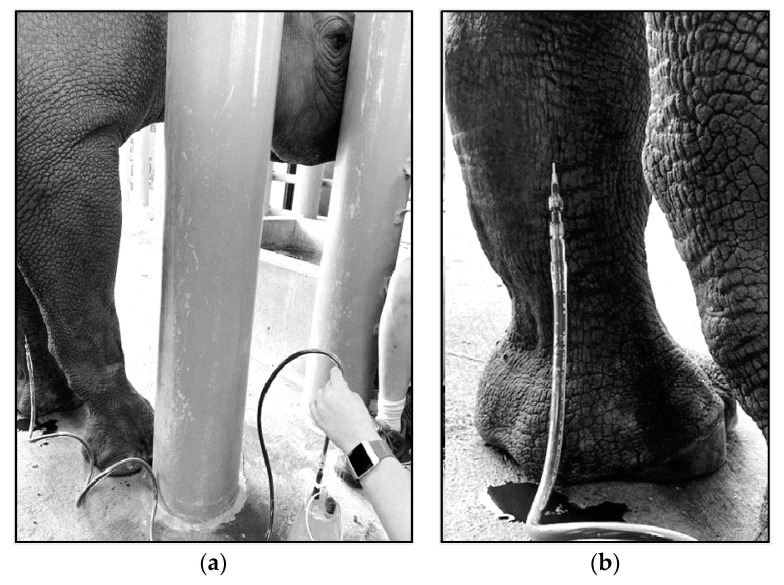
Catheterization of the Radial Vein ((**a**): zoomed out; (**b**): zoomed in). An 18 gauge catheter placed in the right front radial vein; medial forelimb. Anchoring of the catheter is not necessary, and venous access remains patent without securing. Large bore tubing aids in rapid blood flow into the negative-pressure collection containers. Photos were taken in an off-exhibit animal holding area.

To perform VLVP, aseptic techniques should be followed. A certified veterinary technician (CVT) or venipuncture-trained animal keeper should perform the venipuncture for VTLVP. The radial vein (medial forelimb crossing the carpus) and metacarpal vein (lower dorsal hindlimb) are used due to stability and optimal flow (Figure 5). An 18 gauge (ga), 1¼–2 inch intravenous catheter (SURFLO^®^ I.V. Catheter, Terumo Medical Corp., Somerset, NJ, USA, 08873) is commonly used. An 18 ga 1–1¼ inch hypodermic needle can replace the catheter. Catheterization does not require securing or anchoring and is advantageous in that the plastic “over-the-needle” cannula lacks a bevel-edged lumen, which allows it to rest parallel within the vessel with reduced risk of laceration and occlusion along the vessel wall (Figure 6). Large bore tubing (MILA 180 cm blood collection set, MILA International, Inc., Florence, KY, USA, 41042) is connected to the hub of the needle/catheter; the opposite end of the tubing is equipped with an IV spike and drip chamber which is inserted into the rubber stopper on the top of the 1 L glass evacuated collection container (B. Braun Medical, Inc., Bethlehem, PA, USA, 18018). Historically, a 500 mL collection bottle proved ideal, but these are not currently available.

**Figure 7:**
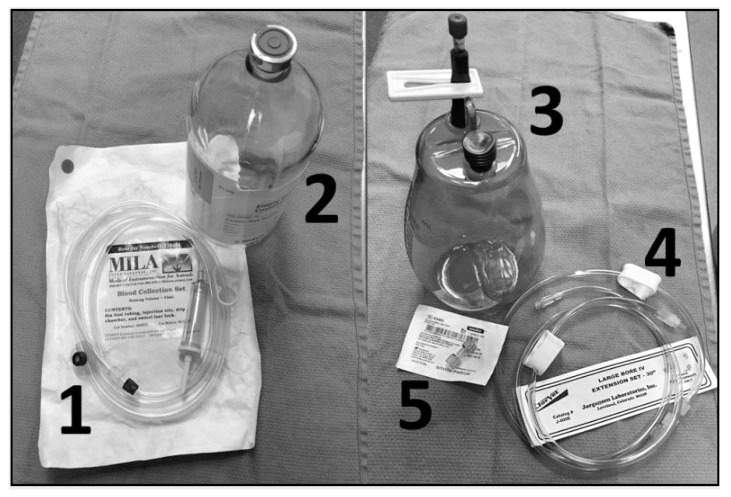
Medical Supplies for Large Volume Blood Collection. Various supply options are available from multiple medical distributors. (**1**) MILA 80” large animal disposable blood collection set with drip chamber (MILA International, Inc., Florence, KY, USA). (**2**) Braun 1000 mL empty glass evacuated container (B. Braun Medical, Inc., Bethlehem, PA, USA). (**3**) Evolution™ UreSil^®^ pre-vac plastic wound drainage bottle (Pacific Hospital Supply Co., Ltd., Miaoli, Taiwan). (**4**) JorVet™ 30” large bore IV extension set (Jorgensen Laboratories, Inc., Loveland, CO, USA). (**5**) Double male Luer lock adapter (Smiths Medical, Dublin, OH, USA).

Specific equipment may need to change based on market availability. Therefore, wound drainage bottles could be used (Evolution™ Pre-vac wound drainage bottle, Pacific Hospital Supply Co., Ltd., Taipei 112, Taiwan), though these lack a rubber stopper and have a female-type connection port; therefore, spike-type IV sets are not compatible. Instead, two large bore IV extension sets (JorVet™ 30” a IV extension set, Jorgensen Laboratories, Inc., Loveland, CO, USA, 80538) connected with a double male Luer lock adapter (Double male Luer lock, Smiths Medical, Dublin, OH, USA, 43017) are required (Figure 7). Using an 18 ga bore, a 75 mL/minute to 100 mL/minute flow rate can be achieved with a standard drip-rate; theoretically, this would increase with pressure (Terumo Medical Corp., Somerset, NJ, USA, 08873). As blood volume reaches the 1 L capacity in a bottle, blood flow slows. The tubing is clamped off, and the full container is exchanged for an empty container until the desired total blood volume is obtained. Observing a decrease in blood flow may indicate occlusion of the tubing or vessel, which should be corrected with slight gentle manipulation of the IV tubing, catheter/needle, or slightly shifting the animal’s position. Fibrin clots, observed as white-pink foamy precipitant in the collection lines, have been noted but do not seem to affect flow rate or collection volumes. After catheter/needle removal, the venipuncture site is held off with manual pressure for several minutes to allow hemostasis and prevention of a hematoma.

As noted above, to improve success, multiple venipuncture sites in different limbs, preferable opposing limbs, have been used in one session. Venipuncture site sensitivity can be mitigated with topical anesthetic cream (lidocaine/prilocaine; EMLA cream 5%, AstraZeneca AB, Södertälje, Sweden), or spray (Gebauer’s ethyl chloride^®^ Mist, Gebauer Company, Cleveland, OH, USA, 44128).

### 4.4. Pharmacologic Chelation Therapy

Due to an animal’s ability to recycle iron, regular phlebotomy is an option for patients with iron-loaded tissues [128]. Other than phlebotomy or pregnancy, the chelation of iron remains the only other option for removing excess iron. The use of synthetic iron chelating agents is crucial in long-term management of certain human forms of iron storage and overload disease. Chelation involves the use of a chemical compound with a high affinity and selectivity for one metal molecule, entering the body, binding that molecule, and clearing both from the body. The ideal chelating ligand for iron has a high binding constant and iron specificity, bioavailability, and forms an inert compound with ferric or ferrous iron [129,130,131]. As there are many forms of chelators (hexadentate, tridentate, etc.), some may bind unintended targets such as other minerals, like zinc or copper, resulting in deficiency. Synthetic chelators have the potential to cause damage to the liver or kidneys, especially when iron or other reactive metals are only loosely bound to the chelator and go through the excretion process [128,132].

Iron chelators bind and target ferric or ferrous iron and can be administered orally, as well as intravenous or intraperitoneal [132]. They target either transferrin bound iron, or non-transferrin bound iron in the plasma, or the labile iron pool (LIP) in the cytoplasm, with very few shown to actively pull iron out of ferritin in the cytoplasm directly [132,133]. Synthetic chelation can result in increased iron clearance from both urinary and fecal routes, dependent on species tested and ligand used [129,134,135,136].

### 4.5. History of Synthetic Chelation for BR

While synthetic iron chelation has been tested in black rhinos, there is limited information at this time supporting safety for widespread use. Synthetic chelators come in a variety of chemical forms with variability in iron mobilization and clearance from the body, as well as the efficacy of the form of administration. Pharmacokinetics are unknown in this species; thus, dosing with any chelator should start conservatively, with a full understanding of iron chelator dynamics in known species. Desferrioxamine was used successfully in one BR, but found it to be impractical due to the high cost, intravenous route, and impractical volume required [119].

An iron specific oral chelator N,N’-Di(2-hydroxybenzyl) ethylenediamine-N,N’-diacetic acid (HBED) was tested in three black rhinos and showed a successful output of iron [23]. Two animals had no negative impact on health from the treatment, but one animal had a recoverable hemolytic event after concluding treatment, likely because chelation abruptly ended as per the protocol for the study. In this case, the animal was heavily iron loaded, and chelation appeared to be very effective, which, speculatively, in combination with unbound iron, low levels of circulating antioxidants, and relative hypophosphatemia resulted in red cell lysis [23]. This reaction may have been mitigated if the animal had been weaned off the chelator. As the HBED treatment was successful, it should still be considered a valid option for the treatment of IOD with the caveat (as with any chelation) that appropriate precautions and monitoring occur as well as a taper if there is a cessation of treatment.

A number of natural oral chelators have had preliminary evaluations with mixed results. Grape pomace has been tested as a form of natural tannin for iron-binding in black rhinos, but other minerals (Cu and Zn) appeared to be impacted in terms of serum values and digestibility, though inconclusive statistically [137,138]. Also, depending on the origin of the pomace, iron content varied greatly and contributed iron to the diet. Anecdotally, rhino keepers at some institutions within the US may brew and feed tea to BR in minor quantities, with no measurements performed to discern impacts on iron absorption for BR. While tea and other liquid iron-binding compounds have been studied in humans, the impact and dose needed to impact BR remain unknown [139,140]. Supplementation of a form of phytic acid (inositol hexaphosphate) is being investigated as an iron chelator in BR, which also may increase circulating phosphorus and alter phosphorus dynamics [141], as shown in horses [142,143] possibly via microbial activity and net absorption in the hindgut. While plant forms of oral iron chelation are seemingly innocuous, side effects are unknown, and health impacts should be studied as nothing replicates the exact iron-binding of natural browse. There is limited knowledge of the exact suite of natural chelators found in the wide variety of browse species utilized by wild BR. Reports range from 32 to 570 species potentially consumed dependent on range and habitat [11,107,144,145]. While condensed tannins were examined in some BR diets, the extent of chelating compounds or iron-binding capacity has not been fully studied in the wild. More research is warranted to illustrate the complexity of wild browse composition.

## 5. Animal Husbandry and Operant Conditioning Practices

A fundamental component of IOD prevention and treatment includes an institution’s dedication and investment in a progressive science-based behavioral husbandry program. This program is important to aid in excellent and close animal observation, access for blood sampling, the capacity to medicate and to perform large-scale phlebotomy. Achieving successful VTLVP requires an animal’s willing participation. An institution that allows time for keepers to form strong relationships with their animals through training will more easily be able to manage the health issues that can arise as a result of this disorder.

In humans, a preventive approach to abating iron overload via therapeutic phlebotomy is more cost-effective than an a posteriori strategy of treating subsequent disorders related to chronic iron overload, including compromised welfare [119]. VTLVP is the gold-standard of treatment for iron overload in humans, dolphins [65], and avian species [65,146,147]. In order to pursue successful medical management via VTLVP, multiple factors must be considered and supported: (1) staff education on IOD and its effects on animals, (2) a strong, proactive behavioral training program to maximize animal cooperation and sustain desired behaviors, and (3) communication and commitment across departments (husbandry, nutrition, research, and the veterinary team) within an institution.

**Figure 8:**
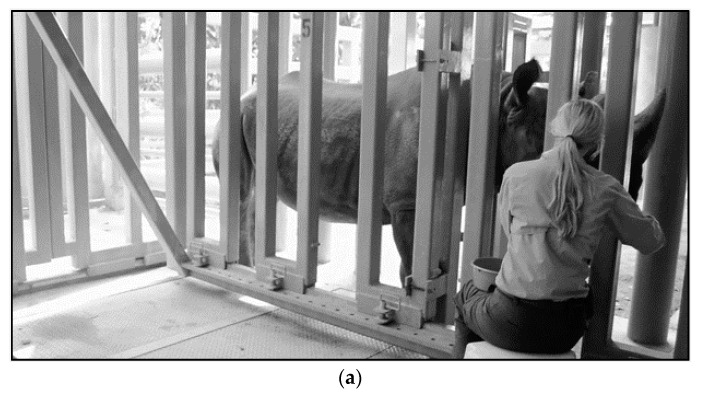

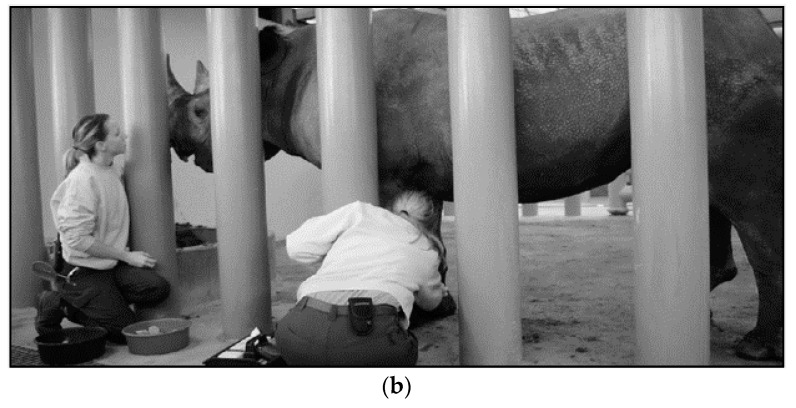
Protected Contact Voluntary Phlebotomy. Proactive behavioral training through operant conditioning for VTLVP has proven successful and reliable (**a**) in chute systems and (**b**) open barn stalls via protected contact. Preference for which training environment is based on individual rhinoceros and the comfort of the animal care staff. Photos were taken in an off-exhibit animal holding area.

**Figure 9:**
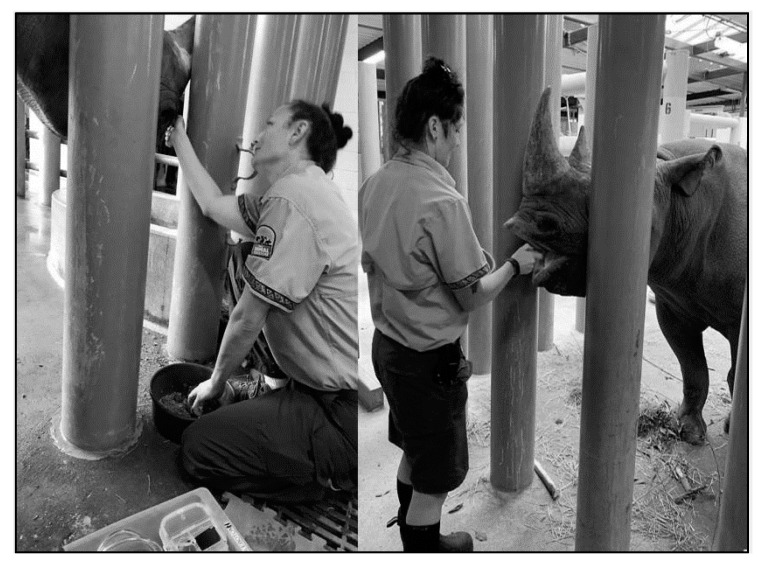
Positive Reinforcement. Reinforcing the rhinoceros to remain calm and still for approximately twenty-five minutes is essential to achieve the desired blood volume. The trainer provides nutritionist-approved dietary items, and tactile attention throughout the session, ensuring the rhino maintains position and is relaxed during the VTLVP process. Photos were taken in an off-exhibit animal holding area.

**Figure 10:**
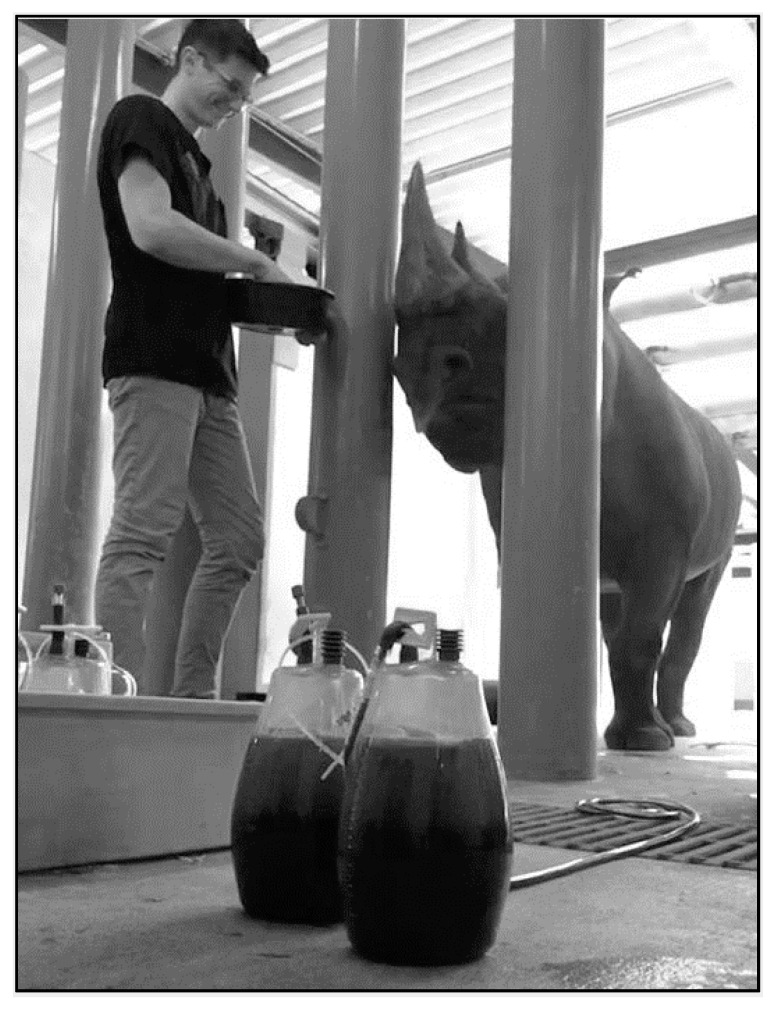
Post-phlebotomy Reinforcement. An adult male BR building a positively reinforced relationship with a certified veterinary technician after a successful VTLVP session. Reinforcing the individual with their preferred diet items helps establish a trusting relationship and promotes future success. Pictured in the foreground are two full containers (1 L each) of blood obtained for quarterly goals. Photo was taken in an off-exhibit animal holding area.

A small, core phlebotomy team (trainers and certified veterinary technicians (CVT)) helps focus training and maintain consistency. Routine positive reinforcement training sessions desensitize the rhinoceros to enter a chute system or stand in an open-barn stall (Figure 8, Figure 9 and Figure 10), which allows the animal staff access to phlebotomy sites. Behavioral training food reinforcers are approved by nutritionists and include items low in iron and vitamin C (see Section 3. Evidence-Based Nutrition Practices). Tactile reinforcement may also be useful during the procedure as a way to maintain the rhino’s interest and cooperation. Appropriate schedules of reinforcement specific to the individual BR, ensuring the animal remains calm and still for at least 25 min, is essential to collect the desired blood volume. Initially, sessions may be as frequent as once or twice per week but can reduce in frequency as the behaviors evolve into a maintenance phase of training. The session would include a combination of behaviors that position the animal, offer target anatomical areas (e.g., foot), allow general desensitization to touch, keep the animal calm, and improve tolerance to larger gauge hypodermic needles. Individual BR may require significant desensitization to the presence of unfamiliar personnel and their attire, unusual smells or sounds (bottles abutting or package material crinkling), and new equipment. Offering a variety of approved food items (i.e., produce, pellets, browse, and bean paste) during VTLVP sessions is important for keeping the BR interested by rotating food items during the session. Physical considerations for animal comfort during the session can include massaging of the legs, placing mats on the ground for padding, application of topical ectoparasite repellants (Endure^®^ sweat-resistant fly spray for horses, Farnam Companies, Inc., Schaumburg, IL, USA, 60173), and realignment for accessibility or squaring off the legs for better balance and thus enhancing blood flow. During colder weather, the application of warm towels or warm water to the legs has been noted to assist in locating the venipuncture sites. Prior to the VTLVP session, the legs should be cleaned with soapy water and/or alcohol to prep the venipuncture sites and reduce the amount of time needed for the session [148].

For animals to tolerate larger needle gauges, a sequential process can be approximated, starting with a generic metal rod (e.g., a paperclip or similar item), dull needles, then small needles (25 ga, then 21 ga), working up to a larger needle (20 ga, 18 ga). Detailed records of venipuncture sites (e.g., leg used, location of the site on the leg, and rotation of leg) are important for later review should a problem arise. VTLVP sessions generally use larger gauge needles than regular blood collection; therefore, it is suggested to have designated areas on the legs for VTLVP and for regular blood collection (i.e., above the carpus (knee) for VTLVP and below the carpus for regular blood collection). There are concerns about vessel scarring (as in human medicine) with repeat venipuncture; however, this has not occurred in this species in the authors’ experience. Possible consequences of repeat venipuncture can include phlebitis (infection), scarring, nerve damage, extravasation (hematoma), and thrombus (clot) formation [149], but the unique anatomy of BR as well as ensuring proper technique minimize negative consequences.

Voluntary participation of BR in VTLVP should be a priority in medical training programs as it reduces the need for immobilization or sedative procedures which require equipment resources, have budgetary impacts, and hold some anesthetic risk. If an animal is sedated or anesthetized, the opportunity to perform LVP is strongly encouraged if there is any evidence of IOD. Erring on the side of caution, LVP should still be considered even without bloodwork immediately prior to sedation since it is likely that the individual has some degree of iron accumulation. Other beneficial applications of maintaining VTLVP behaviors include blood donation for transfusions, plasma banking for medically compromised individuals, and voluntary participation in other medical treatments (e.g., vaccine administration) and other zoo research studies requiring blood.

## 6. Conclusions

**Figure 11:**
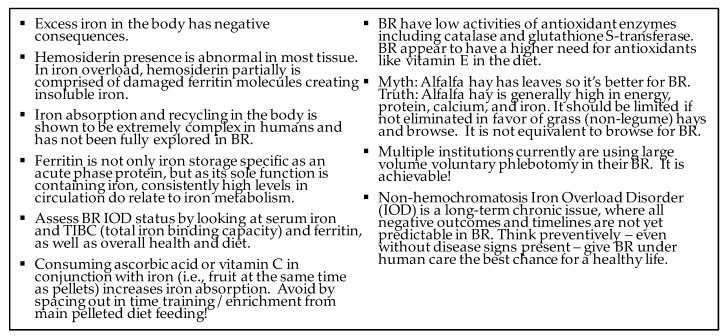
Highlights of important points and common misconceptions related to iron balance and BR management.

Non-hemochromatosis Iron Overload Disorder requires long-term monitoring for diagnosis, prevention, and treatment. Given the survival of BR as a species is dependent on successful reproduction in zoos, it is a priority to abate iron overload [2,70,150,151]. Taking action to think holistically and preventively in approaching this long-term pervasive disorder can serve to help maintain healthier BR (Figure 11). The Center of the Study of Iron in Rhinos (CSI-R) hopes to continue collaborating and supporting our rhino community to move knowledge forward while sharing best practices.

The “take home messages” for managing IOD in BR to consider are:

(1) Evaluate iron-related blood parameters to help understand baseline and then status over time and in relation to other health measures (Figure 2).
(2) Focus attention on diet and nutrition, especially in terms of limiting iron.
(3) Treat animals when indicated, preferably early in disease course; consider treatment options and guidance from experts on phlebotomy and potential chelation.
(4) Invest time in training BR to allow for routine sampling as well as potential treatments. While it seems challenging, it is accomplishable without significant equipment or changes in stall design.
(5) Balance the high cost of not assessing or managing IOD in BR with the low cost of using available tools to help guide treatments that can be very effective and prolong the life of the animal, improve reproductive success, and reduce disease issues, all of which have immeasurable costs.

## Figures and Tables

**Table 1:** Moisture (%), dry matter (DM;%), iron (ppm DM), and vitamin C (mg/100 g as fed; AF), calcium (%DM), and phosphorus (%DM) values in example diet items commonly used for black rhinoceros. Feed composition can be quite variable depending on harvest location, manufacturer, and season. Nutrients included in the table are not sufficient to balance an animal’s diet or to evaluate inclusion as training or enrichment food items; thus, it is recommended to consult a nutritionist. For example, although bananas are lower in iron and vitamin C than cauliflower, bananas are high in starch (~23% DM) and thus should be limited in favor of metabolic health. Another cautionary example is appreciating the high moisture in produce such as leafy greens, which dilutes nutrient concentrations in the AF product. Thus, produce items generally are not used to balance nutrient concentrations such as calcium or phosphorus.

	Moisture	Dry Matter	Iron	Vitamin C	Calcium	Phosphorus
Food Item	%	%	ppm (DM) *	mg/100 g (AF) **	(%DM)	(%DM)
**Pelleted feed examples:**						
Mazuri ADF 25 Herbivore diet	10.5	89.5	652	nd	1.51	0.95
Mazuri ADF 16 Herbivore diet	8.2	91.8	490	nd	1.16	0.84
Mazuri Browser Rhino Cube 5Z1P	10.4	89.6	222	nd	1.22	0.68
**Produce examples:**						
Cucumber (raw, whole)	97.6	2.4	86	3.2	0.89	1.35
Carrot (raw, whole)	88.9	11.1	35	5.9	0.31	0.25
Celery (raw, whole)	95.1	4.9	29	3.1	0.98	0.51
Sweet potato (raw, whole)	79.8	20.2	22	2.4	0.36	0.29
Apple (raw, whole)	88.0	12.0	11	4.6	0.05	0.08
**Produce with higher vitamin C or iron level examples:**						
Green Leaf lettuce (fresh, raw)	94.9	5.1	278	9.2	0.74	0.66
Spinach (fresh, raw)	91.4	8.6	264	28.1	1.02	0.73
Romaine (fresh, raw)	95.6	4.4	152	11.5	0.73	0.67
Green beans (fresh, raw)	92.2	7.8	101	12.2	0.55	0.53
Cantaloupe melon (fresh, whole)	93.1	6.9	74	36.7	0.11	0.17
Cauliflower (raw, whole)	94.0	6.0	60	48.2	0.55	0.75
Tomatoes (raw, whole)	95.7	4.3	53	13.7	0.18	0.47
Honeydew melon (fresh, whole)	91.6	8.4	37	18.0	0.11	0.33
Watermelon (fresh, whole)	92.3	7.7	33	8.1	0.14	0.33
Banana (raw, whole with peel)	82.4	17.6	15	8.7	0.07	0.12
Orange (raw, whole with peel)	82.6	17.4	14	71.0	0.57	0.13
**Hay/Fresh Browse examples:**						
Alfalfa Hay	12.0	88.0	386	nd	1.84	0.31
Coastal Bermudagrass Hay	10.0	90.0	52	nd	0.57	0.19
Timothy Hay	11.0	89.0	48	nd	0.54	0.09
Mulberry (whole branch fresh)	67.6	32.4	84	nd	1.7	0.38
Willow (whole branch fresh)	63.2	36.8	63	nd	0.77	0.13
Spineless cactus pads (Opuntia)	91.5	8.5	22	nd	3.67	0.17
**Supplement examples:**						
Dicalcium Phosphate	4.6	95.4	12,200	nd	23.4	19.7
Trace mineral block	0.1	99.9	1790	nd	0.3	0.05
Dried Beet Pulp with molasses	7	93	731	nd	1.15	0.1
Molasses	8.0	92.0	577	0.0	0.18	0.02
Wheat bran	8.4	91.6	186	nd	0.15	1.48
Steamed Rolled Oats	8.5	91.5	41	0.0	0.05	0.48

* Dry matter, moisture, calcium, phosphorus, and iron determined on feed at Disney’s Animal Kingdom^®^, analyzed by Dairy One Laboratories (Ithaca, NY). ** Vitamin C values sourced from the USDA database (https://www.nal.usda.gov/usda-food-composition-database). “nd” indicates the nutrient value for the food item was not determined.

**Table 2:** Individual BR (BR 1, BR 2) average quarterly voluntary therapeutic large volume phlebotomy (VTLVP) volumes taken per session at Disney’s Animal Kingdom^®^ (DAK). Averages based on several (2–3) sessions within a quarter (Q).

VTLVP at DAK: Average Quarterly Volumes								
**BR 1**	**2013**	**2014**	**2015**	**2016**	**2017**	**2018**	**2019**	**2020**
**Q1 (January–March)**	10.0 L	5.5 L	7.0 L	4.0 L	3.7 L	4.0 L	1.0 L	4.0 L
**Q2 (April–June)**	4.0 L	3.0 L	6.0 L	3.5 L	4.0 L	4.0 L	0.5 L	NA
**Q3 (July–September)**	5.5 L	0.5 L	0.0 L	4.0 L	0.0 L	4.0 L	4.0 L	4.0 L
**Q4 (October–December)**	7.5 L	3.0 L	2.5 L	0.0 L	0.0 L	4.0 L	3.0 L	-
**BR 2**	**2013**	**2014**	**2015**	**2016**	**2017**	**2018**	**2019**	**2020**
**Q1 (January–March)**	NA	NA	NA	NA	6.0 L	2.0 L	2.0 L	2.0 L
**Q2 (April–June)**	NA	NA	NA	NA	3.0 L	3.0 L	3.0 L	NA
**Q3 (July–September)**	NA	NA	NA	NA	2.0 L	4.0 L	4.0 L	0.0 L
**Q4 (October–December)**	NA	NA	NA	NA	1.0 L	2.0 L	4.0 L	-

“NA” indicates that no VTLVP was performed.
